# Anticoagulation Patterns in Patients with Atrial Fibrillation Following Percutaneous Coronary Intervention in an Academic Center

**DOI:** 10.19102/icrm.2019.100802

**Published:** 2019-08-15

**Authors:** Akash Rusia, Kenneth Y. Kita, Shawn D. Shah, Dipayon Roy, Youning Zhang, Jonathan Nattiv, Natasha Doshi, Han Tun, Leonardo C. Clavijo, Rahul N. Doshi

**Affiliations:** ^1^Keck School of Medicine, University of Southern California, Los Angeles, CA, USA

**Keywords:** Anticoagulation, atrial fibrillation, coronary intervention, dual antiplatelet therapy

## Abstract

A common dilemma facing physicians treating patients with atrial fibrillation (AF) who have undergone percutaneous coronary intervention (PCI) is the management of oral anticoagulation (OAC) therapy, because there is also an indication for dual antiplatelet therapy in these patients. The purpose of this study was therefore to evaluate anticoagulation patterns in this patient population in an attempt to identify patterns of risk factors that may influence OAC prescribing habits. This retrospective study entailed a review of a total of 4,648 patients from two academic hospitals who underwent PCI between 2008 and 2016. We ultimately included 211 patients who had AF and an indication for OAC. Chart review revealed patients’ risk factors, CHA_2_DS_2_-VASc and HAS-BLED scores, and antithrombotic regimens. Only 105 (49.8%) patients who met the indications for OAC were actually placed on OAC post-PCI. There was no significant relationship between discharge on OAC and HAS-BLED score (t = 0.14; p = 0.44) or CHA_2_DS_2_-VASc score (t = 0.76; p = 0.22). Patients younger than 65 years of age were prescribed more triple therapy (56% versus 33%; p < 0.01) or any OAC (69% versus 41%; p < 0.01) on discharge in comparison with patients 65 years of age or older. The older patient group had a significantly higher average CHA_2_DS_2_-VASc score (4.4 versus 3.2; p < 0.01) and a higher average HAS-BLED score (2.8 versus 2.4; p < 0.01). Ultimately, this study indicated that less than half of AF patients with an indication for OAC were placed on OAC post-PCI. There was no association between discharge on OAC and CHA_2_DS_2_-VASc score, HAS-BLED score, or any other individual risk factor, with the exception of age.

## Introduction

Atrial fibrillation (AF) is the most common sustained arrhythmia,^1^ and thromboembolic prophylaxis is a mainstay in the clinical management of its most debilitating complication—stroke. Meanwhile, percutaneous coronary intervention (PCI) is one of the most commonly performed cardiovascular procedures, with more than 500,000 PCI procedures performed annually in the United States alone.^2^ As a result, a common dilemma faced by many physicians is the management of AF and oral anticoagulation (OAC) in post-PCI patients who also require dual antiplatelet therapy (DAPT).

A lack of strong evidence and consensus regarding the optimal combination of therapies has led to many different philosophies and treatment strategies, including some based on CHA_2_DS_2_-VASc and HAS-BLED scores, which stratify thromboembolic risk and bleeding risk, respectively.^3,4^ The 2013 What is the Optimal Antiplatelet and Anticoagulant Therapy in Patients With OAC and Coronary Stenting (WOEST) trial provided some insight into the safety of the two strategies by randomizing patients with AF undergoing PCI to triple therapy (OAC and DAPT) versus double therapy (OAC and clopidogrel).^5^ Results from this investigation indicated that double therapy was associated with a significant reduction in bleeding complications and led to a class IIb recommendation in the 2014 American Heart Association/American College of Cardiology/Heart Rhythm Society (AHA/ACC/HRS) AF guidelines, where it was noted that it may be reasonable to use double instead of triple therapy in this specific patient group.^6^

Several other studies including Safety and Efficacy of Six Months Dual Antiplatelet Therapy After Drug-eluting Stenting (ISAR-SAFE) and Triple Therapy in Patients on OAC After Drug-eluting Stent Implantation (ISAR-TRIPLE) have suggested that a shorter duration of DAPT after PCI may be acceptable and could help to reduce bleeding events.^7,8^ In 2018, a meta-analysis comparing double and triple antithrombotic therapy in patients with AF who underwent PCI found roughly similar rates of thromboembolism but a 47% increase in thrombolysis in myocardial infarction major or minor bleeding for patients receiving triple therapy.^9^ Although a number of investigations have attempted to identify a regimen that can maximize thromboembolic and stent thrombosis prophylaxis while minimizing bleeding risk, the lack of corroborating evidence has largely left physicians with underwhelming guidance.

Regardless of the data presented, however, practitioners and patients alike are biased in their decision-making when it comes to anticoagulation in AF.^10^ This leads to a phenomenon of suboptimal prescription of OAC in patients with AF^11^ despite overwhelming evidence demonstrating associated benefits.^12–17^ Given the suboptimal rate of anticoagulation of AF patients on the whole, it is reasonable to suspect that the subset of patients who are also undergoing PCI would similarly have a suboptimal rate of anticoagulation.

In the present research, we reviewed the prescribing habits for patients with AF undergoing PCI to dissect the potential reasons for this and to identify patterns of risk factors that may influence anticoagulation prescribing habits. Our hypothesis was that there would be a correlation between stroke OAC prescription and risk, bleeding risk, or certain other risk factors.

## Methods

From January 2008 to April 2016, 4,648 PCIs were performed at the Los Angeles County and University of Southern California Medical Center and the Keck Hospital of the University of Southern California. A retrospective chart review was performed for each patient with special attention paid to medications and problem lists on admission and discharge. This evaluation revealed 224 patients with AF undergoing PCI and appropriate documentation of their antithrombotic regimen on admission and discharge. We then were able to create a database of these patients including their demographics, medication lists, and risk factors.

The CHA_2_DS_2_-VASc score was calculated for each of the 224 patients to establish indications for OAC. Thirteen patients did not meet indications for anticoagulation and were given a score of less than two points. Of the remaining 211 patients, a HAS-BLED score was calculated to assess bleeding risk. The antithrombotic regimen of each patient on discharge was evaluated to assess for correlation with CHA_2_DS_2_-VASc score, HAS-BLED score, individual thromboembolic risk factors, or antithrombotic regimen prior to PCI, respectively. An independent samples t-test was conducted to compare CHA_2_DS_2_-VASc and HAS-BLED scores for discharge regimens with and without OAC. A chi-squared test was used to assess the relationship between discharge antithrombotic regimen and each individual risk factor.

## Results

**Figure 1:**
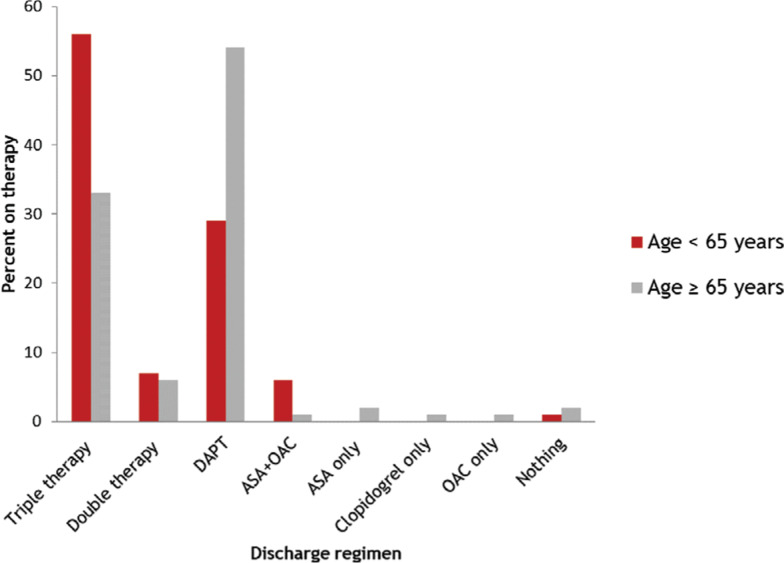
Antithrombotic regimen in patients with AF undergoing PCI (n = 211). Overall, 46.0% of patients were discharged with DAPT, while 40.3% were discharged with triple therapy. When stratified by age, there were significantly more patients younger than 65 years of age who were prescribed triple therapy or any regimen that included OAC as compared with patients older than 65 years of age. Expectedly, the reverse was true for DAPT: patients who were older than 65 years of age were prescribed significantly more DAPT than those who were younger than 65 years of age. ASA: aspirin; DAPT: dual antiplatelet therapy; OAC: oral anticoagulation.

Of the 211 AF/PCI patients who met indications for OAC, 105 (49.8%) were placed on OAC post-PCI. Overall, 97 (46.0%) patients were placed on DAPT only, 85 (40.3%) were given triple therapy, and 19 patients (9.0%) were given a combination of OAC and a single antiplatelet agent **(Figure 1)**. Patient characteristics and demographics are summarized in **Table 1**. Of the patients who received OAC, six specifically were placed on direct OAC (DOAC) therapy.

**Figure 2:**
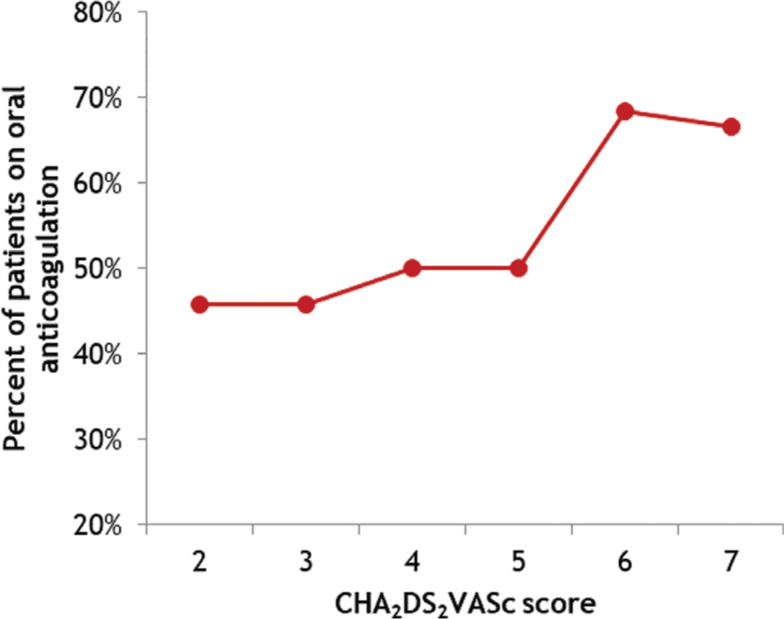
Percentage of patients with AF undergoing PCI who were prescribed oral anticoagulation on discharge for each CHA_2_DS_2_-VASc score.

There was no significant difference between discharge regimens with and without OAC for either CHA_2_DS_2_-VASc scores (4.05 ± 1.30 versus 3.91 ± 1.40; t = 0.76; p = 0.22) or HAS-BLED score (2.69 ± 1.11 versus 2.71 ± 1.12; t = 0.14; p = 0.44). These results suggest that neither CHA_2_DS_2_-VASc score **(Figure 2)** nor HAS-BLED score have an effect on whether or not patients were discharged on OAC. Regarding the individual risk factors, no association was found between discharge on OAC and congestive heart failure, systemic hypertension, vascular disease, female gender, or diabetes mellitus **(Table 2)**. Although there was a trend toward association, a history of stroke or transient ischemic attack was also not significantly associated with discharge on OAC (p = 0.14).

Patients who were younger than 65 years of age (average age: 57.9 years) were more likely to be anticoagulated than patients who were 65 years of age or older (average age: 76.2 years). Furthermore, the younger patients were prescribed more triple therapy (56% versus 33%; p < 0.01) or any OAC (69% versus 41%; p < 0.01) on discharge. Conversely, the older patient group had a significantly higher average CHA_2_DS_2_-VASc score (4.4 versus 3.2; p < 0.01) and a significantly higher HAS-BLED score (2.8 versus 2.4; p < 0.01). Expectedly, if a patient was admitted on OAC, they were more likely to be discharged with OAC (p < 0.01).

## Discussion

The most feared and debilitating complication of nonvalvular AF is stroke. The rate of stroke in this population averages approximately 5% per year, or two to seven times the rate in those without nonvalvular AF.^18^ The mainstay of stroke risk management remains anticoagulation; however, the inherent risk of bleeding is a concern that has consistently led to the inconsistent management of cerebrovascular accident (CVA) risk. Nowhere is this more evident than in those patients with AF who are post-PCI and who require DAPT and OAC.

The current study has shown that only half of the study participants who met the indication for OAC for AF following PCI were actually placed on OAC. We found no correlation when we delved into whether clinical risk factors such as a history of CVA, CHA_2_DS_2_-VASc score, or HAS-BLED score correlated with the decision to discharge patients on OAC. In fact, the only two factors that had a statistically significant association with OAC on discharge were whether a patient was already on OAC prior to PCI and if they were younger than 65 years of age. These findings are in line with what’s expected in a community of physicians who lack a consensus on how to manage such individuals. Moreover, these findings seem to confirm that physicians continue to base decisions on clinical gestalt rather than on strong data supporting OAC in the context of either triple or double therapy. This is due in part to weak direction from guidelines.

Our study found that younger AF patients had a lower stroke risk and were more likely to be anticoagulated than older AF patients, which confirms prior findings.^19^ A review by Bungard et al. in 2000 found that physicians generally base their decision to anticoagulate older AF patients on the perceived risk of embolism and hemorrhage.^10^ Some surveyed physicians, however, noted that they have withheld anticoagulation in patients based on the belief that patients may refuse treatment or may not be compliant, although there is evidence that, when given the choice, most patients would comply with treatment.^20^ Nevertheless, a reduced stroke risk generally far outweighs the risk of major hemorrhage while on anticoagulation and most concerns about bleeding are generally unfounded.^21^ Simply put, the oldest patients with AF are those who have the highest risk of stroke and the most to gain from anticoagulation. While the risk of falls is in many cases the documented reason for withholding anticoagulation, several analyses have shown that warfarin still has a significant net protective effect against composite outcomes of out-of-hospital death or hospitalization for stroke, myocardial infarction, or hemorrhage.^22^ Evidence points to focusing on maintaining international normalized ratio (INR) values of 2.0 to 3.0 (therapeutic range) and ensuring proper blood pressure control as a means of mitigating intracranial hemorrhage risk, as opposed to withholding anticoagulation.^23,24^

Our data were not powered enough to detect a difference in prescribing habits between warfarin and DOACs, as only six of the 105 patients who received OAC received a DOAC specifically. We have seen an increase in the use of DOACs as compared with warfarin; some studies have even shown a resulting increase in the overall percentage of AF patients receiving OAC.^25^ A meta-analysis of four existing major randomized control trials for DOACs showed a significant reduction in stroke, intracranial hemorrhage, and mortality (albeit with increased gastrointestinal bleeding) in comparison with warfarin.^26^ In real-world use, compliance rates are generally similar between warfarin and DOACs; however, warfarin requires INR monitoring, which allows physicians to directly see where noncompliance may be an issue.^27^ The highest thromboembolic risk appears to be among patients who are not compliant with DOAC therapy. Nevertheless, with more AF patients being prescribed anticoagulation in the post-DOAC era, it will be of interest to see whether long-term rates of thromboembolism show strong improvement.

The findings of this study were based on patient data from 2008 to 2016; however, in more recent years, landmark trials have shown that treatment with OAC and a P2Y12 inhibitor may perhaps be safer and noninferior in terms of efficacy.^5,6,9^ Thus, a limitation of this study relates to the fact that trends in prescribing habits are expected to change with double therapy regimens being increasingly utilized. This study was also limited due its retrospective design and the quality of the data retrieved from electronic records being beholden to the accuracy of the documenting physician. Future studies in this arena could also elicit more explicit information as to why providers chose one regimen over another or why patients were not able to tolerate OAC; this may provide a clearer picture of the reasons for the suboptimal prescription of OAC in patients with AF.

## Conclusions

Less than half of patients who met indications for OAC for AF after PCI were discharged on appropriate therapy. Patients were more likely to be discharged on OAC if they were already on OAC at the time of admission or if they were younger than 65 years old. There was no association between discharge on OAC and CHA_2_DS_2_-VASc score, HAS-BLED score, or any other individual risk factor, with the exception of age. This study ultimately presents three observations, as follows: (1) there are major gaps in treating physicians’ perceptions of the scientific consensus on how to treat AF patients undergoing PCI; (2) there is a lack of established guidelines to direct community physicians; and (3) there remains a stigma regarding treating older patients with anticoagulation.

## Figures and Tables

**Table 1:** Patient Characteristics

Characteristic		Total Patients (n = 211)	Discharge Regimen		
			Any Regimen with OAC [n = 105 (49.8%)]	No OAC [n = 106 (50.2%)]	Triple Therapy [n = 85 (40.3%)]
Average age, years		70.3 ± 11.0	68.0 ± 11.6	72.6 ± 10.0	68.0 ± 11.6
	< 65 years	68 (32.2)	47 (44.8)	21 (19.6)	38 (44.7)
Age, n (%)	65–75 years	72 (34.1)	27 (25.7)	45 (42.1)	22 (25.9)
	> 75 years	71 (33.6)	31 (29.5)	40 (37.4)	25 (29.4)
Female gender, n (%)		45 (21.0)	20 (19.0)	25 (23.4)	16 (18.8)
Heart failure, n (%)		120 (56.0)	61 (58.7)	59 (55.7)	47 (55.3)
Hypertension, n (%)		171 (81.0)	87 (82.9)	84 (79.2)	69 (81.2)
Diabetes mellitus, n (%)		122 (57.8)	59 (56.2)	63 (59.4)	46 (54.1)
Stroke/TIA, n (%)		27 (12.8)	17 (16.2)	10 (9.4)	15 (17.6)
Vascular disease, n (%)		117 (55.5)	56 (53.3)	61 (57.5)	45 (52.9)
CHA_2_DS_2_-VASc score		4.0 ± 1.4	4.0 ± 1.3	3.9 ± 1.4	4.1 ± 1.3
HAS-BLED score		2.7 ± 1.1	2.7 ± 1.1	2.7 ± 1.1	2.7 ± 1.1

OAC: oral anticoagulation; TIA: transient ischemic attack.

**Table 2:** Patients on Oral Anticoagulation with Presence or Absence of Risk Factors

Risk Factor	Patients with Risk Factor in Question Who Were Prescribed OAC	Patients Without Risk Factor in Question Who Were Prescribed OAC	p-value
Congestive heart failure	51%	48%	0.72
Hypertension	51%	45%	0.50
Vascular disease	48%	52%	0.53
Diabetes mellitus	48%	52%	0.55
Prior stroke or TIA	63%	48%	0.14
Age < 65 years	69%	41%	0.01

OAC: oral anticoagulation; TIA: transient ischemic attack.
